# Condensed Milk as Infants' Food

**Published:** 1920-09-11

**Authors:** 


### Condensed Milk as Infants' Food.

In his quarterly report the Hull City public analyst refers to condensed milk. Samples examined were in the main of normal composition, though one labelled as "full cream" contained only 7.2 per cent. of milk fat, and must be regarded as of suspiciously poor quality. In the case of three samples the label directs that the product should be diluted with an equal quantity of water, which would result in the production of a milk containing four per cent. of fat. Such diluted milk is quite good enough for general kitchen use and for use with tea, etc., but it contains too much added sugar (twenty per cent. or more) for infant feeding; and digestive troubles are bound to occur if such products are habitually given to infants. If the original condensed milks are further diluted, as recommended by the canners of the two other samples examined, in respect of which one part of milk is directed to be diluted with four or five, or with seven or eight, parts of water, too little milk fat is present in the final product to make it suitable diet for infants, and although the sugar is reduced to a reasonable amount, the milk fat is lowered to two per cent., or even less. In the opinion of the analyst sweetened condensed milk cannot be made suitable for use in infant feeding, but in all cases where artificial feeding must be resorted to there is probably nothing better than dried milk put up in tins. This product is uniform in composition, clean, and of high nutritive value. Four samples of such dried milk examined during the quarter contained over twenty-five per cent. of milk fat and were free from preservatives and other objectionable additions. It is interesting to note that the Ministry of Health has appointed a committee to consider the question of condensed milk. No condensed milk should be labelled "full cream" unless its content of fat is at least ten per cent., for the consumer is accustomed to add water, whatever the concentration.

